# IL-13 induces periostin and eotaxin expression in human primary alveolar epithelial cells: Comparison with paired airway epithelial cells

**DOI:** 10.1371/journal.pone.0196256

**Published:** 2018-04-19

**Authors:** Yoko Ito, Reem Al Mubarak, Nicole Roberts, Kelly Correll, William Janssen, James Finigan, Rangnath Mishra, Hong Wei Chu

**Affiliations:** Department of Medicine, National Jewish Health, Denver, Colorado, United States of America; University of Alabama at Birmingham, UNITED STATES

## Abstract

Alveolar epithelial cells are critical to the pathogenesis of pulmonary inflammation and fibrosis, which are associated with overexpression of type 2 cytokine IL-13. IL-13 is known to induce the production of profibrotic (e.g., periostin) and pro-inflammatory (e.g., eotaxin-3) mediators in human airway epithelial cells, but it remains unclear if human primary alveolar epithelial cells increase periostin and eotaxin expression following IL-13 stimulation. The goals of this study are to determine if alveolar epithelial cells increase periostin and eotaxin expression upon IL-13 stimulation, and if alveolar and airway epithelial cells from the same subjects have similar responses to IL-13. Paired alveolar and airway epithelial cells were isolated from donors without any lung disease, and cultured under submerged or air-liquid interface conditions with or without IL-13. Up-regulation of periostin protein and mRNA was observed in IL-13-stimulated alveolar epithelial cells, which was comparable to that in IL-13-stimulated paired airway epithelial cells. IL-13 also increased eotaxin-3 expression in alveolar epithelial cells, but the level of eotaxin mRNA was lower in alveolar epithelial cells than in airway epithelial cells. Our findings demonstrate that human alveolar epithelial cells are able to produce periostin and eotaxin in responses to IL-13 stimulation. This study suggests the need to further determine the contribution of alveolar epithelial cell-derived mediators to pulmonary fibrosis.

## Introduction

Fibrotic lung diseases such as idiopathic pulmonary fibrosis (IPF) remain a significant challenge in clinical care, and highlight the need of more rigorous basic and translational research to define their pathogenesis and effective therapy [1–3]. Previous studies suggest that type 2 cytokines, particularly IL-13, are involved in the inflammatory and fibrotic processes [4, 5]. In airways diseases such as asthma, IL-13 has been shown to increase the expression of periostin and eotaxins (e.g., eotaxin-3) that are the key mediators in airway remodeling and eosinophilic inflammation [6, 7]. Interestingly, eosinophilic inflammation also exists in pulmonary fibrosis, and has been used as a marker of disease progression [8]. Periostin is a matricellular protein that is expressed by many types of cells, particularly in diseased conditions [9, 10]. It has multiple functions, including tissue repair and remodeling (e.g., fibrosis). Various cytokines such as IL-13 and TGF-beta act on structural and immune cells to promote periostin production [11, 12].

Among the tissue structural cells involved in IPF, alveolar epithelial cells play a critical role [13, 14]. During lung injury, type I alveolar (ATI) cells are injured, which are then repaired and replaced by type II alveolar (ATII) cells, leading to restoration of alveolar structure [15]. However, under pathological conditions, ATI cells may not be repaired, which in turn promotes persistent injury and remodeling with fibrosis as the most common but deleterious outcome [16].

The regulation of periostin and eotaxins has been extensively studied in human large airway epithelial cells exposed to IL-13 [17–19]. Interestingly, eotaxins promote fibroblast migration, a key process of fibrosis [20]. However, whether periostin is up-regulated in alveolar epithelial cells by IL-13 is unclear. Moreover, whether alveolar and airway epithelial cells respond similarly to IL-13 stimulation regarding periostin and eotaxin expression has not been addressed. By leveraging a cell bank of paired primary human alveolar and airway epithelial cells at our institution, we performed a cell culture study to determine IL-13-mediated periostin and eotaxin-3 expression by alveolar epithelial cells. The use of paired samples overcame the concern of genetic determinants in comparing the results obtained from alveolar and airway epithelial cells. Unraveling the role of alveolar epithelial cells in terms of periostin and eotaxin expression likely improves our understanding of periostin and/or eotaxin as biomarkers of lung injury/fibrosis predicting disease activity/severity and therapeutic responses.

## Materials and methods

### Materials

Recombinant human IL-13 protein from the R&D Systems, Minneapolis, MN was reconstituted in 0.1% bovine serum albumin (BSA) and stored at -80°C. Bronchial epithelial cell growth medium (BEGM) with antibiotics was purchased from Lonza, Walkersville, MD, USA. Air-liquid interface culture media (F6 media) consisted of 1:1 ratio of bronchial epithelial basal medium (BEBM) and Dulbecco's modified eagle medium (DMEM) plus insulin, transferrin, epinephrine, bovine pituitary extract (BPE), gentamicin and amphotericin, bovine albumin (0.5 μg/ml, ethanolamine (80 μM), MgCl_2_ (0.3 mM), MgSO_4_ (0.4 mM), CaCl2 (1 mM), retinoic acid (30 ng/ml), hEGF (10 ng/ml). Bovine collagen I (3 mg/ml) was obtained from Advanced BioMatrix (San Diego, CA). RNA lysis buffer (RLT) was from Qiagen (Hilden, Germany). RIPA Western lysis buffer was purchased from Thermo-Fisher Scientific (Waltham, MA).

### Human donor information

To isolate human primary ATII and airway epithelial cells, we obtained human lungs from de-identified organ donors whose lungs were not suitable for transplantation and donated for medical research through the National Disease Research Interchange (Philadelphia, PA), the International Institute for the Advancement of Medicine (Edison, NJ) or Donor Alliance of Colorado. The Institutional Research Board (IRB) at National Jewish Health approved this study (approval number: HS-2598). Donors were chosen based on lung function with a Pa_O2_/FiO2 ratio of >225, no history of clinical lung diseases, a chest radiograph indicating no infection, and a time on the ventilator of <5 days. Table 1 shows the clinical information of five donors included for our current study who did not have any lung disease.

**Table 1:** Characteristics of donors included in the study.

Subjects	Age (yrs)	Gender	Smoking status	Cause of death
1	30	Female	Smoker (1/2 pack for 15 yrs)	Anoxia
2	52	Male	Smoker	Cerebrovascular accident (CVA)
3	44	Male	Smoker (1/2 pack for 30 yrs)	CVA
4	54	Male	Smoker (1/2 pack for 30 yrs)	Anoxia
5	18	Female	Non-smoker	Anoxia

### Isolation and culture alveolar epithelial cells

We modified the human primary ATII cell isolation method published by Fang et al. [21]. Briefly, the right middle lobe of the lung was perfused, lavaged, and then instilled with elastase (Worthington, Lakewood, NJ) for 40 min at 37°C. The lung was minced, and the cells were isolated by filtration and partially purified by centrifugation on a discontinuous density gradient made of Optiprep (Accurate Chemical Scientific, Westbury, NY) with densities of 1.080 and 1.040, and by negative selection with binding to IgG-coated Petri dishes (Sigma, St. Louis, MO). The isolated cells were resuspended in DMEM supplemented with 10% FBS, 2 mM L-glutamine, 2.5 μg/ml amphotericin B, 100 μg/ml streptomycin, 100 μg/ml penicillin G (GIBCO, Life Technologies, Rockville, MD), and 10 μg/ml gentamicin (Sigma-Aldrich, St. Louis, MO).

For submerged culture, human primary ATII cells (2.0x10^6^ cells/well) were plated on 12-well cell culture plates (Costar, Corning, NY) coated with rat-tail collagen (Thermo Fisher Scientific, Waltham, MA) in DMEM with 10% FBS (D10). On day 2, the culture medium was changed to DMEM with 5% FBS (D5) with recombinant human IL-13 protein (rhIL-13,10 ng/ml) (R&D systems, Minneapolis, MN) or 1% BSA (vehicle for rhIL-13). Cell lysates and culture medium were harvested at 24 or 48 hrs after stimulation.

For air-liquid interface (ALI) culture, human primary ATII cells (1.5x10^6^ cells/well) were plated on Millicell hanging inserts (pore size 1.0 μm, membrane surface area 1.1 cm^2^) (EMD Millipore, Darmstadt, Germany) in 12 well plates coated with a mixture of 20% Matrigel (Corning Inc., Corning, NY) and 80% rat-tail collagen in D10 (apical 0.5 ml, basal 1.5 ml). On day 2 and day 4, the culture medium was changed to DMEM with 1% charcoal-stripped (CS) FBS (Thermo Fisher Scientific), 2 mM L-glutamine, 2.5 μg/ml amphotericin B, 100 μg/ml streptomycin, 100 μg/ml penicillin G, 10 μg/ml gentamicin, 10 ng/ml recombinant human keratinocyte growth factor (R&D systems) (KGF) and 10^−8^ M dexamethasone (Dex) designated as 1%CS with KGF and Dex (apical 50 μl, basal 1.2 ml). On day 6 the culture medium was changed to 1%CS with KGF and Dex with 10 ng/ml rhIL-13 or 1% BSA. Then, cell lysates and culture medium were harvested at 24 or 48 hrs after stimulation.

Because the data from 24 and 48 hrs were similar, we have presented the data from the 48 hour time point in the Results section.

### Isolation and culture of human tracheobronchial airway epithelial cells

To isolate tracheobronchial epithelial (TBE) cells, tracheal and main bronchial tissue was digested with 0.1% protease in DMEM (GE Life Sciences, Logan, Utah) overnight at 4°C, and processed as previously described [22]. TBE at passage one were expanded in collagen-coated 60-mm tissue culture dishes in BEGM media at 37 °C, 5% CO_2_. When the cells were 80% confluent, they were trypsinized and seeded onto collagen-coated 12-well plates for submerged culture and transwell inserts for ALI cultures.

For submerged culture, TBE cells in BEGM media were seeded into 12-well plates (2x10^5^ cells/well) coated with collagen. After 48 hours of seeding, the medium was changed, followed by IL-13 stimulation (10 ng/ml) or 0.1% BSA treatment. The cells were harvested after 24 and 48 hrs in RLT or in RIPA buffer with protease and phosphatase inhibitors. The supernatants were collected for ELISA.

**Fig 1:**
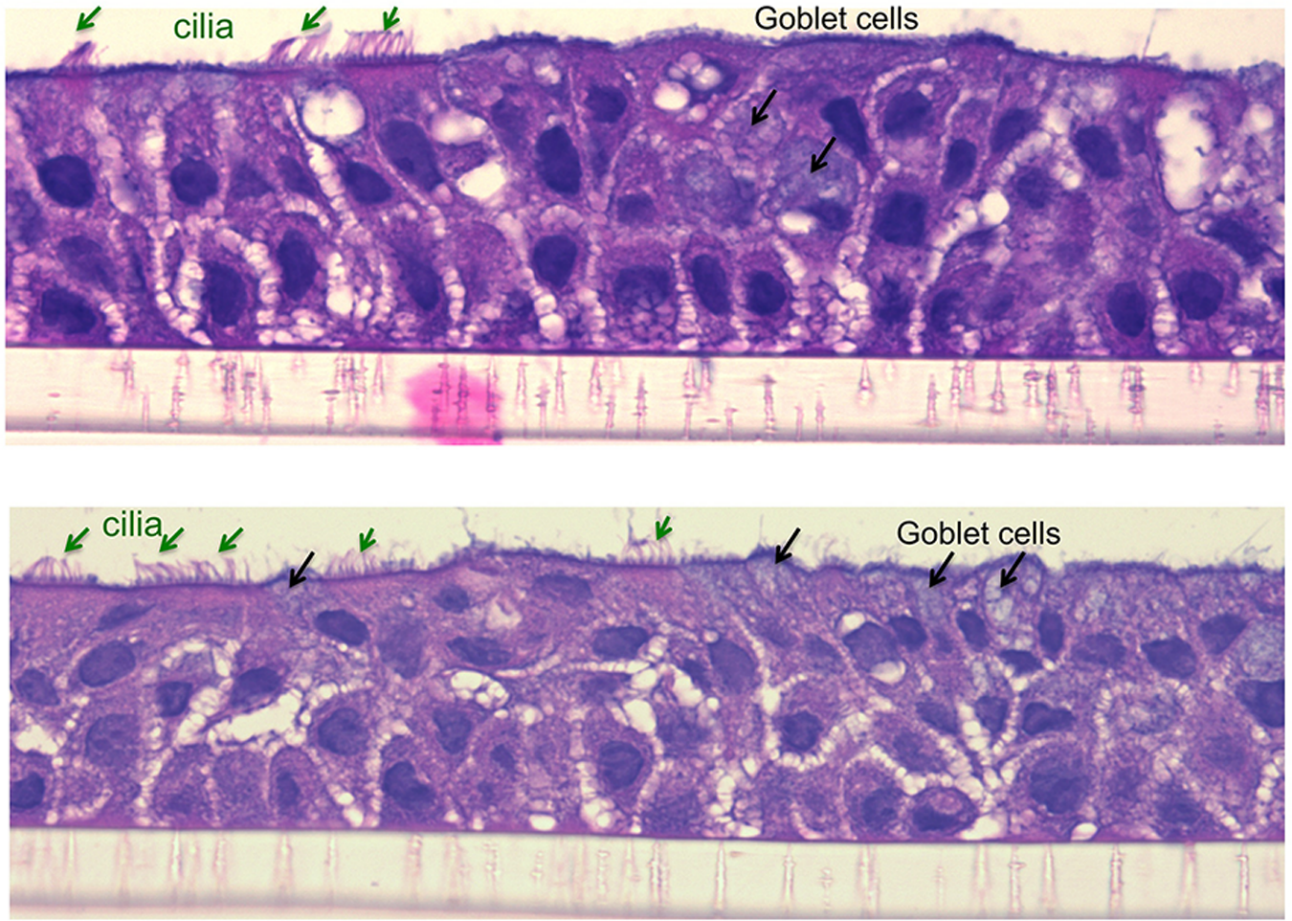
Mucociliary differentiation of human tracheobronchial epithelial (TBE) cells grown at the air-liquid-interface culture for 14 days. H&E staining, original magnification x400.

For ALI culture, TBE cells were seeded at 4x10^4^ cells/insert onto collagen coated transwell inserts in 12-well plates with 300 μL of F6 media at apical surface, and 1.2 mL of F6 media at the basolateral side. After a week in the immersed culture condition, the cells reached 100% confluence and were shifted to ALI condition by reducing the apical medium volume to 50 μL. ALI conditions were maintained for 14 days when cells demonstrated mucociliary differentiation (Fig 1), and treatment with IL-13 (10 ng/ml) or 0.1% BSA started on day 15. The cells and the apical and basolateral media were harvested at 24 and 48 hrs post IL-13 treatment.

Because the data from 24 and 48 hrs were similar, we have presented the data from the 48 hour time point in the Results section.

### ELISA of human periostin and eotaxin-3

Periostin and eotaxin-3 protein levels were measured in cell supernatants using their specific DuoSet ELISA kits (R&D Systems, Minneapolis, MN) as per manufacturer’s instruction.

### RT-PCR for human periostin and eotaxin-3

Real-time PCR was performed on the CFX96 (Bio-Rad) using TaqMan gene expression assays purchased from the Applied Biosystems (Life Technologies, Foster City, CA, USA). An identical threshold was applied to each gene of interest. mRNA relative expression levels were calculated using the delta cycle threshold (Ct) method. Target gene (e.g., periostin) expression was normalized to GAPDH.

### Western blot analysis

Equal amounts of total proteins from samples with different treatments were separated on 15% SDS-PAGE, transferred onto polyvinylidene difluoride (PVDF) membranes, and probed with an anti-proSP-C antibody (Seven Hills Bioreagents, Cincinnati, OH), an anti-NKX2.1 antibody (R&D Systems, Minneapolis, MN), an anti-AQP5 antibody (Sigma-Aldrich, St. Louis, MO) or a mouse anti-ß-actin (Santa Cruz Biotechnology, Inc.). Blots were then incubated with appropriate HRP-linked secondary antibodies and developed with ECL Western blot substrate.

### Immunoflurorescent staining of ATII cells

To confirm ATII differentiation of alveolar epithelial cells cultured at air-liquid interface, cells on Transwell membranes were fixed in 10% formalin, embedded in paraffin, and cut at 5 μm thickness for immunoflurescent staining. Briefly, cell sections were incubated with a mouse anti-human AT II cell primary antibody (provided by Dr. L. G. Dobbs, University of California, San Francisco), followed by incubation with a secondary antibody (goat anti-mouse IgM) conjugated with Texas red. After the cells were washed, DAPI was added to counterstain the nuclei. The cells were coverslipped and viewed under a fluorescence microscope.

### Statistical analysis

The Mann-Whitney test was used for data comparisons of the two groups. A p value < 0.05 was considered significant.

## Results

### Alveolar epithelial cells increase periostin and eotaxin-3 expression upon IL-13 stimulation

**Fig 2:**
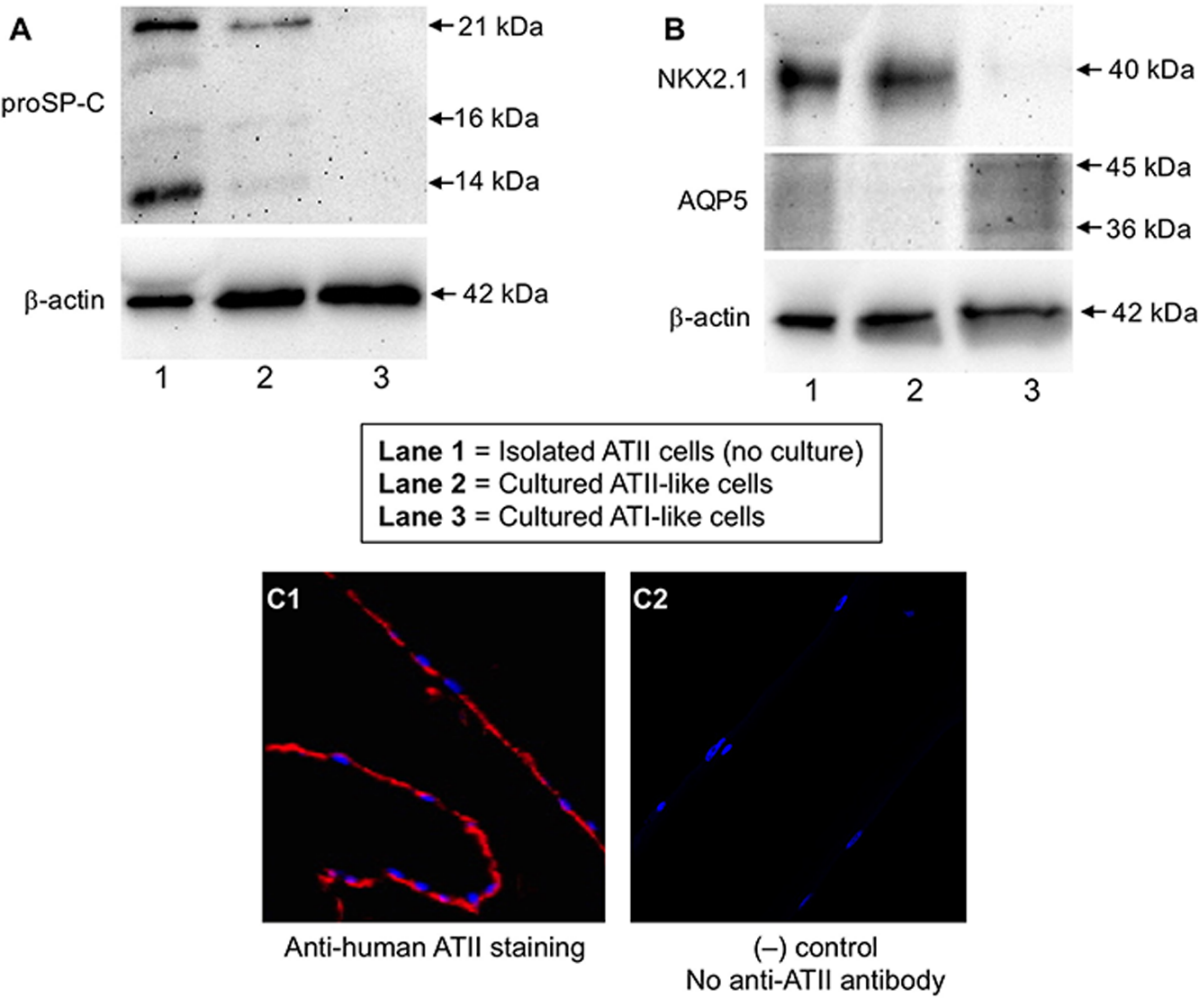
Confirmation of alveolar type II (ATII)-like cells cultured at the air-liquid interface. Alveolar epithelial cells isolated from a donor were cultured for 7 days to differentiate into ATII-like cells. The cultured ATII-like cells, isolated (non-cultured) ATII cells (positive control) and cultured AT1-like cells from the same donor were processed for Western blot of proSP-C (**A**), NKX2.1 and AQP5 (**B**). Additional bands were observed for pro-SP-C (predicted size: 21 kDa) and AQP5 (predicted size: 36 kDa), which is likely due to post-translational modifications, post-translational cleavages, and/or other experimental factors. Cells on the transwell membrane were embedded in paraffin and cut into a 5 μm thickness section for immunofluorescent staining of ATII-like cells (**C1**) using an anti-ATII cell antibody as described in the Methods section. **C2**: the negative control—no addition of an anti-ATII cell antibody during immunofluorescent staining. The red and blue colors indicate the cytoplasm and nuclei of ATII-like cells, respectively.

**Fig 3:**
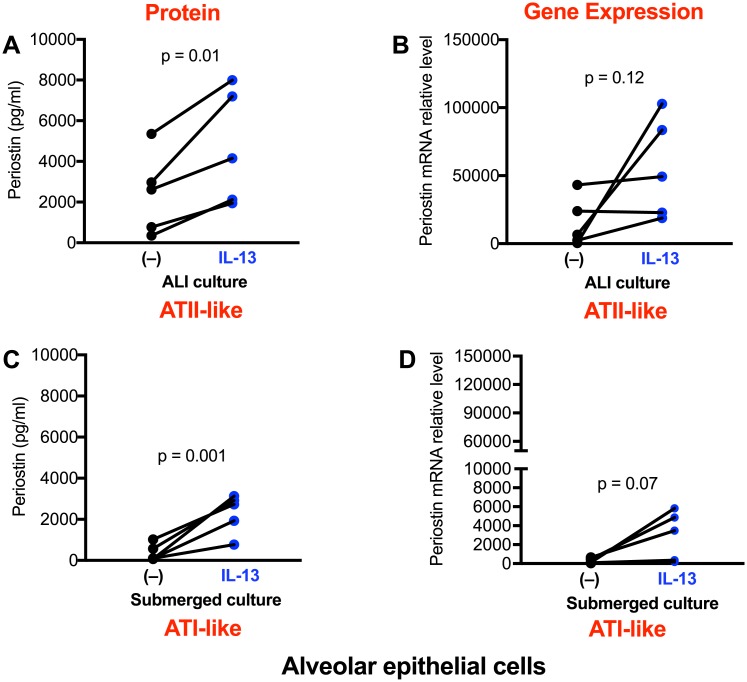
Alveolar epithelial cells increase periostin expression after IL-13 treatment. Cells under the air-liquid interface (**A, B**) and submerged (**C, D**) cultures were stimulated with IL-13 for 48 hrs. Periostin protein and mRNA were measured by ELISA and real-time RT-PCR, respectively. N = 5 donors.

**Fig 4:**
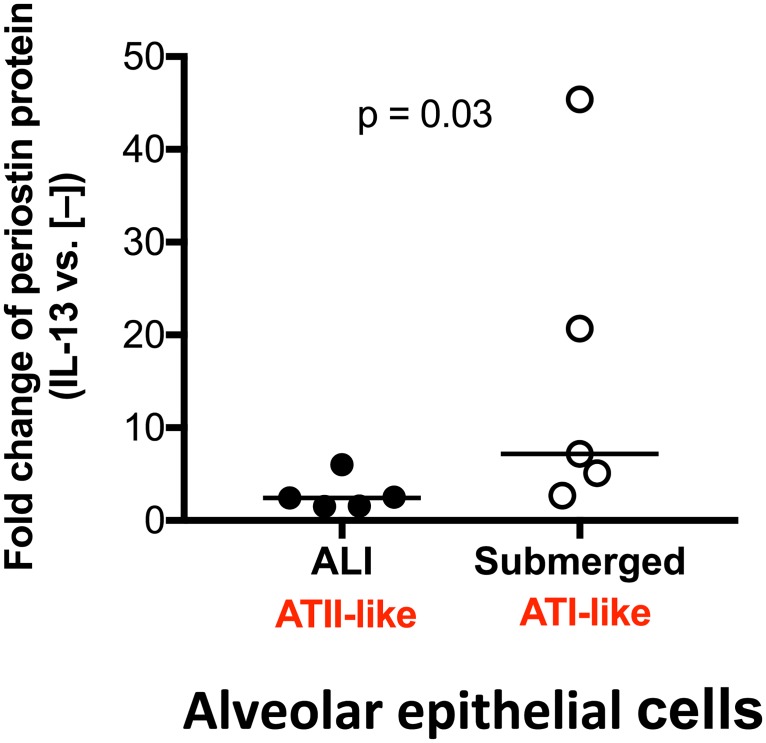
Comparison of the fold changes of periostin protein induction by IL-13 stimulation in alveolar epithelial cells cultured under air-liquid interface (ALI) versus submerged conditions. The horizontal bars indicate the medians. N = 5 donors.

Under ALI culture, alveolar epithelial cells underwent ATII-like differentiation as confirmed by Western blot of proSP-C and NKX2.1, and immunofluorescent staining for ATII cells (Fig 2A, 2B and 2C). As shown in Fig 3A, alveolar epithelial cells cultured in ALI produced modest levels of periostin in basolateral supernatants. Periostin increased significantly following IL-13 stimulation. Periostin mRNA was also increased by IL-13, but was not statistically significant (p = 0.12) (Fig 3B). Under submerged culture, alveolar epithelial cells present an ATI-like phenotype as they expressed AQP5, but not proSP-C and NKX2.1 (Fig 2A and 2B). Like the ALI culture data, IL-13 increased periostin protein secretion (Fig 3C) and levels of periostin mRNA (Fig 3D), however the mRNA increase was not statistically significant. Of note, ATI-like cells expressed lower levels (14.2-fold, p = 0.008) of periostin mRNA than the ATII-like cells in the presence of IL-13 stimulation. However, the fold changes of periostin protein induction by IL-13 stimulation were greater (about 3-fold, p = 0.03) in ATI-like cells than ATII-like cells (Fig 4).

**Fig 5:**
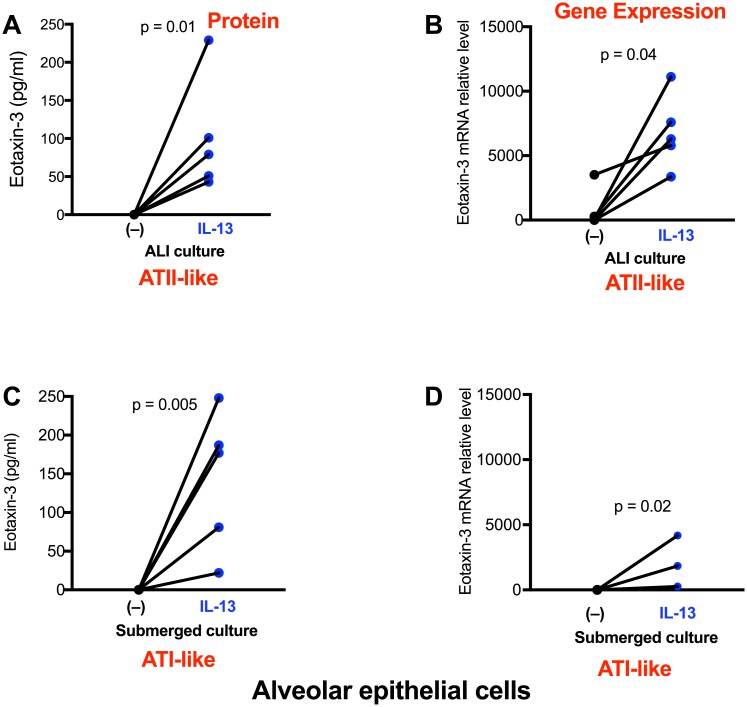
Alveolar epithelial cells increase eotaxin-3 expression after IL-13 treatment. Cells under the air-liquid interface (**A, B**) and submerged (**C, D**) cultures were stimulated with IL-13 for 48 hrs. Eotaxin-3 protein and mRNA were measured by ELISA and real-time RT-PCR, respectively. N = 5 donors.

Alveolar epithelial cells also demonstrated an increase in eotaxin-3 protein and mRNA expression (p<0.05) following the IL-13 treatment under both ALI and submerged conditions (Fig 5A to 5D). Upon IL-13 stimulation, eotaxin-3 mRNA levels in AT1-like cells were lower (3.4-fold, p = 0.02) than those in the ATII-like cells.

### Comparison of IL-13-induced periostin and eotaxin-3 expression between alveolar epithelial cells and tracheobronchial epithelial (TBE) cells

IL-13 has been shown to induce periostin and eotaxin-3 in airway epithelial cells. However, the relative abundance of periostin and eotaxin expression in paired airway and alveolar epithelial cells from the same subjects has not been compared.

**Fig 6:**
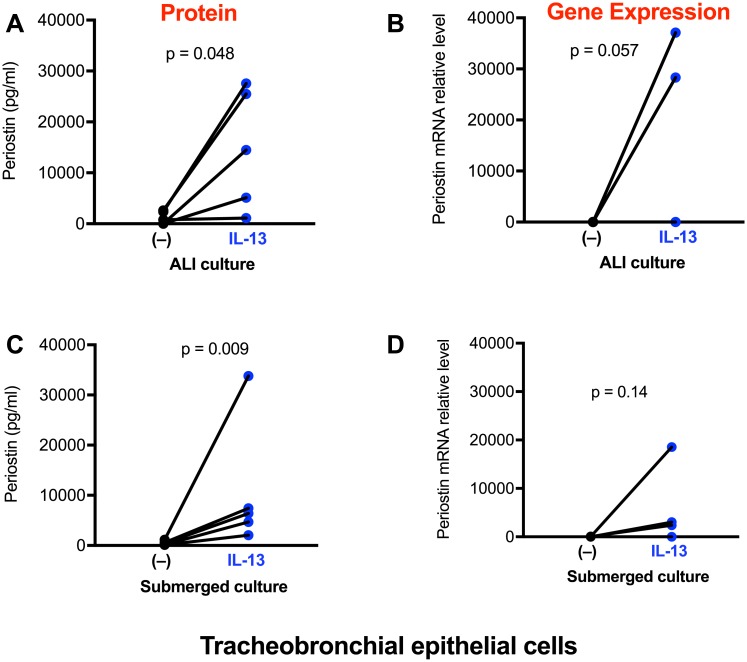
Tracheobronchial epithelial (TBE) cells increase periostin expression after IL-13 treatment. Cells under the air-liquid interface (**A, B**) and submerged (**C, D**) cultures were stimulated with IL-13 for 48 hrs. Periostin protein and mRNA were measured by ELISA and real-time RT-PCR, respectively. N = 5 donors.

Under ALI condition, IL-13 consistently increased periostin protein production in TBE cells and the induced increase was statistically significant (Fig 6A). Although IL-13 in TBE cells increased periostin mRNA, there was no significant difference between the controls and IL-13-stimulated cells (Fig 6B). Notably, in the presence or absence of IL-13 stimulation, periostin mRNA levels trended to be lower (about 3-fold, p = 0.09) in well-differentiated TBE cells than ATII-like cells. Due to the differences of cell density and components of cell culture media between alveolar and airway epithelial cells, comparison of periostin proteins levels was not performed.

Under submerged culture, IL-13 also increased periostin protein expression in TBE cells (Fig 6C). However, the increase in periostin mRNA levels by IL-13 was not statistically significant (Fig 6D). Both TBE and alveolar epithelial cells demonstrated similar levels of periostin mRNA.

**Fig 7:**
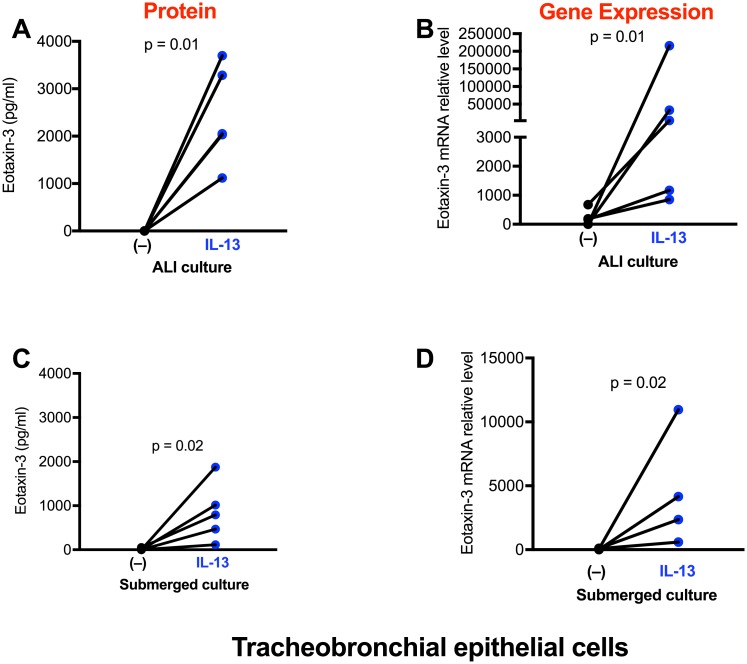
Tracheobronchial epithelial (TBE) cells increase eotaxin-3 expression after IL-13 treatment. Cells under the air-liquid interface (**A, B**) and submerged (**C, D**) cultures were stimulated with IL-13 for 48 hrs. Eotaxin-3 protein and mRNA were measured by ELISA and real-time RT-PCR, respectively. N = 5 donors.

As expected, TBE cells expressed very low levels of eotaxin-3 mRNA and protein in the absence of IL-13 stimulation (Fig 7A to 7D). After IL-13 treatment, TBE cells significantly increased eotaxin-3 expression. TBE cells under ALI culture expressed higher levels of eotaxin-3 mRNA than cells under the submerged condition. Interestingly, unlike the periostin mRNA data, eotaxin-3 mRNA expression induced by IL-13 under ALI culture was similar (p = 0.84) between TBE cells and alveolar epithelial cells

## Discussion

This is the first cell culture study to examine the expression of periostin and eotaxin in alveolar epithelial cells isolated from humans. We found that like the airway epithelial cells, alveolar epithelial cells increase periostin protein expression following IL-13 treatment. Notably, ATII-like cells expressed higher levels of periostin mRNA than ATI-like cells. Since periostin may contribute to fibrosis, our study may provide a rationale to inhibit periostin expression in alveolar epithelium under diseased conditions such as pulmonary fibrosis.

Examining the effects of IL-13 on alveolar epithelial periostin expression is significant. Alveolar epithelial cell injury and repair are critical to the pathogenesis of interstitial lung diseases such as pulmonary fibrosis. In this regard, ATII cell dysfunction is a common feature of fibrotic lungs [23]. Periostin expression is elevated in lung tissue and blood of patients with IPF [24, 25], however it remains unclear if alveolar epithelial cells contribute. Although ATII cells produce various profibrotic cytokines such as TGF-beta [26, 27], it is unknown whether they produce periostin at the normal and diseased conditions. We discovered that even at the baseline, ATII-like cells do express a detectable level of periostin mRNA and protein. The physiological relevance of periostin expression by “normal” ATII cells has not been investigated. Given the beneficial effect of periostin on wound repair [28], a “normal or physiological” level of periostin would be critical to maintain the alveolar structure and function. However, a pathological level of periostin, such as that significantly induced by IL-13, would be detrimental to the lung injury and repair. As periostin is able to induce fibroblast differentiation into myofibroblasts, and bind to other extracellular matrix components [9], targeting its aberrant up-expression would be pivotal to more effectively control the fibrotic process. Our results suggest the need to target alveolar epithelial cell-derived periostin in lungs exposed to type 2 cytokine IL-13.

The results from study on submerged culture of alveolar epithelial cells allow us to determine how normal ATI-like cells respond to IL-13 stimulation. Interestingly, ATI-like cells express much lower levels of periostin mRNA than ATII-like cells. These findings suggest that the ATI cells are likely less involved in the fibrotic process.

It is well established that IL-13 induces periostin in human primary airway epithelial cells [29], which may in part explain the profibrotic role of IL-13 in the airways. It is unclear whether IL-13-mediated periostin production is similar between alveolar and airway epithelial cells. By using the paired alveolar and airway epithelial cells from the same donors, we demonstrated that both types of epithelial cells robustly increase periostin expression following IL-13 treatment. Moreover, airway basal cells (submerged culture) and mucociliary cells (air-liquid interface culture) demonstrated a similar increase of periostin mRNA upon IL-13 stimulation. Together, when IL-13 is increased in the lung, the entire lung epithelial system, whether intact or injured, would respond by increasing periostin expression. As periostin has multiple functions involved in repair, fibrosis and inflammation [9], it remains to be determined if up-regulation of periostin by IL-13 in airway vs. alveolar epithelial cells would result in similar or different outcomes.

An additional finding in this study is to demonstrate the ability of alveolar epithelial cells to increase eotaxin expression upon IL-13 treatment. Although eosinophilia is a common feature in asthmatic airways, it also exists in the alveolar tissue of patients with pulmonary fibrosis [30–32]. Our results suggest that alveolar epithelial cells may contribute to eosinophil recruitment into fibrotic lungs under a type 2 cytokine (e.g., IL-13) milieu. Comparison of eotaxin-3 mRNA expression in paired alveolar and airway epithelial cells indicate that airway and alveolar epithelial cells respond similarly to IL-13. This finding further suggests the ability of entire lung epithelium to induce eosinophilic inflammation. Future studies are warranted to determine if a subset of pulmonary fibrosis patients with elevated type 2 cytokines such as IL-13 may increase eotaxin production coupled with worse fibrosis and clinical outcomes.

We are aware of several limitations in the current study. First, the cells we used were from donors without any lung disease. As there is no published evidence showing perisotin expression by ATII cells in IPF patients, it would be important to compare periostin and eotaxin expression levels in alveolar and airway epithelial cells isolated from lungs of patients with or without pulmonary fibrosis. Second, we used cells from both smokers and a non-smoker. As direct exposure of cultured lung epithelial cells to cigarette smoke affects eotaxin and periostin expression [33, 34], future studies are warranted to determine if smoking history has a significant impact on IL-13-mediated periostin and eotaxin expression in human airway and alveolar epithelial cells. Third, the varying baseline expression of periostin between ATI-like and ATII-like cells may be explained in part by the use of different media for submerged and air-liquid interface cultures of alveolar epithelial cells. Lastly, the functions of perisotin up-regulation by IL-13 were not determined in the context of epithelial cells and fibroblasts. We plan to utilize gene over-expression or knockdown approaches to reveal the impact of periostin on epithelial or fibroblast proliferation, secretion of surfactant proteins, mucins or collagens.

In summary, our research findings suggest that like the airway epithelial cells, alveolar epithelial cells represent one of the major sources of periostin in a type 2 inflammation setting. This work would set the foundation for future exploration of alveolar epithelial cells-derived periostin in various physiological and pathological processes.
